# The well-being of community-dwelling near-centenarians and centenarians in Hong Kong a qualitative study

**DOI:** 10.1186/1471-2318-14-63

**Published:** 2014-05-13

**Authors:** Wai-Ching Paul Wong, Hi-Po Bobo Lau, Chun-Fong Noel Kwok, Yee-Man Angela Leung, Man-Yee Grace Chan, Wai-Man Chan, Siu-Lan Karen Cheung

**Affiliations:** 1 Department of Social Work and Social Administration, Hong Kong SAR, China; 2 Sau Po Centre on Ageing, Hong Kong SAR, China; 3 Centre for Suicide Research and Prevention, Hong Kong SAR, China; 4 Department of Psychology, Hong Kong SAR, China; 5 Department of Nursing, The University of Hong Kong, Hong Kong SAR, China; 6 Hong Kong Council of Social Service, Hong Kong SAR, China; 7 The Department of Health, Hong Kong SAR, China

**Keywords:** Ageing, Hong Kong SAR, Chinese culture, Community and public health, Health and well-being, Psychology, Psychosocial issues

## Abstract

**Background:**

Hong Kong has one of the highest life expectancy rankings in the world. The number of centenarians and near-centenarians has been increasing locally and internationally. The relative growth of this population is a topic of immense importance for population and health policy makers. Living long and living well are two overlapping but distinct research topics. We previously conducted a quantitative study on 153 near-centenarians and centenarians to explore a wide range of biopsychosocial correlates of health and “living long”. This paper reports a follow-up qualitative study examining the potential correlates of “living well” among near-centenarians and centenarians in Hong Kong.

**Methods:**

Six cognitively, physically, and psychologically sound community-dwelling elders were purposively recruited from a previous quantitative study. Semi-structured interviews were conducted.

**Results:**

Four major themes related to living long and well emerged from the responses of the participants: (a) *Positive relations with others*, (b) *Positive events and happiness*, (c) *Hope for the future*, and (d) *Positive life attitude*. Specifically, we found that having good interpersonal relationships, possessing a collection of positive life events, and maintaining salutary attitudes towards life are considered as important to psychological well-being by long-lived adults in Hong Kong. Most participants perceived their working life as most important to their life history and retired at very old ages.

**Conclusions:**

These findings also shed light on the relationships between health, work, and old age.

## Background

The worldwide population is ageing at an unprecedented rate [1]. It is projected that the proportion of the world population aged 80 or above will increase almost seven-fold by 2100, from 120 million in 2013 to 392 million in 2050, and to 830 million in 2100 [2]. The number of centenarians will remarkably increase by about 18 times, from 180,000 in 2000 to 3.2 million by 2050 [1]. In Hong Kong, the number of centenarians has also increased four-fold, from 289 in 1981 to 1,890 in 2011 (about 3/10,000, [3]). With a current population of about 7.1 million, the oldest-old (80+) segment is expected to grow at a rate more than twice that of young- to mid-old (age 60-79) (2.7%) [4], and expand from about 246,100 in 2010 (3.5% of the total population) to 956,800 in 2041 (12%) [3].

Many researchers have attempted to examine why and how near-centenarians and centenarians live to such an advanced age. Examples of some long-standing centenarian studies include The Longitudinal Study of Danish Centenarians [5-7], The Okinawa Centenarian Study [8], The US Georgia Centenarian Study [9,10], The US New England Centenarian Study [11,12], and The Chinese Longitudinal Healthy Longevity Study (CLHLS) [13]. Most studies have focused on discussing the biological aspects and family history, with less than one-third of them, however, having examined psychosocial aspects of living long [10].

Several studies have underscored the importance of psychosocial mechanisms for understanding how very long-lived adults maintain their quality of life despite increasing constraints [14,15]. Jopp and Rott [16] found that happiness was robustly predicted by extroverted personality, social network, and self-referent beliefs. In addition, von Faber et al. [17] found that the predictive efficacy of physical health and cognitive astuteness was overshadowed by the abovementioned psychosocial factors. Third, Poon and colleagues [18] discussed the importance of four psychosocial predictors of quality of life including (a) demographics, positive/negative life events and personal history, (b) personality, (c) cognitive functioning, and (d) social and economic resource adequacy. Current knowledge of how psychological aspects contribute to the quality of life and “living well” among centenarians is still sparse.

Qualitative studies afford a great degree of flexibility and individualization in the process of data collection and are therefore well-suited to capture the versatile and complex interactions between participants and the social and physical environment. Moreover, if qualitative interviews are to be conducted in the participants’ residential settings, contextual and environmental information can also be collected and used to enhance the understanding of the interconnections of the potential variables that may and may not lead to longevity. Among the paucity of qualitative studies on exceptionally long-lived adults in the literature [19-23], work ethic, optimism, and large social networks have been found to be the most significant psychosocial correlates.

The present qualitative study included six physically and psychologically healthy community-dwelling participants who were purposively selected based on six different idiographic examinations (i.e., a centenarian living alone; a centenarian living with spouse; a centenarian living with child (ren); a centenarian living with grandchild (ren); a centenarian living with relatives, and a centenarian living with family members and/or domestic helper) using maximum variation sampling. The six participants were chosen from a larger sample of 153 elderly participants in a quantitative study completed in September 2011 by the research team. The detailed methodology of the quantitative study has been reported [4]. Letters of invitation for participation in the quantitative study were sent to day care centers, district elderly community centers, neighborhood elderly centers, social centers for the elderly, home support teams throughout the territory, and the University of the 3rd Age (U3A) centers^a^ to invite eligible elders. The objective of the quantitative study was to explore the characteristics of a range of correlates for healthy longevity, including physical and mental health, functioning dependence (mobility, activities of daily living (ADL) and instrumental activities of daily living (IADL)), current health and biological condition, vision and hearing, the capacity of physical performance, self-rated health, self-evaluation on life satisfaction, demographic attributes, family structure, socioeconomic status, behavioral risk factors, social and family support, health and elderly care service needs in the community, etc. The present qualitative study serves as a follow-up to the quantitative part, and aims to gain a deeper insight into the potential psychological factors that lead to “living well” among centenarians in Hong Kong.

## Methods

The interviews were semi-structured and conducted by three interviewers including the principal investigator (trained demographer), a co-investigator (clinical psychologist), and a psychology PhD candidate, approximately 1 year after the quantitative data collection period. The interviews examined psychological well-being of the participants and were organized around six areas of investigation: *Relations with others*, *Life events*, *Life attitude*, *Hope*, *Happiness*, and *Suggestions to others on achieving longevity*. The interviews attempted to elicit autobiographical reflections and short narratives on participants’ experiences and everyday life both in the past and at present. The interview questions are listed in Appendix A. Key themes were identified based on the six-factor model of psychological well-being proposed by Ryff and Keyes [24], and related literature [17,20,21,23]. The length of each interview ranged from 1 to 1.5 hours. No multiple or proxy interviews took place. All of the six respondents had relatively good self-reported physical health, cognitive functioning (i.e., mini-mental state examination (MMSE) scores ranged from 20 to 30, Mean = 26.33, SD = 3.98), and psychological well-being (i.e., 15-item Geriatric Depression Scale (GDS) score ranged from 0 to 2, Mean = 0.67, SD = 0.82) at the time of the interviews. Cataracts were found to be the most common chronic illness, affecting three of the six participants, while two had hypertension. Gout, thyroid disease, fractures, and osteoporosis also affected some participants (Table 1). All participants reported being able to perform Katz’s [25] six ADL and most of Lawton & Brody’s [26] IADL, except two participants reported that they were not able to travel independently on public transport.

**Table 1:** Characteristics of the participants

**Name**	**A**	**B**	**C**	**D**	**E**	**F**
**Year of birth**	1914	1911	1903	1909	1912	1914
**Gender**	M	F	F	M	F	M
**Place of birth**	China	China	China	China	China	China
**Living status**	Alone	Alone	Alone	Family	Family	Family
**MMSE (Total score = 30)**	27	20	29	29	23	30
**GDS (Total score = 15)**	0	2	1	1	0	0
**Subjective health**	Very good	Fair	Fair	Good	Fair	Very good
**Katz**’**s ADL (6 items)**						
Activities unable to perform independently	None	None	None	None	None	None
**Lawton & Brody**’**s IADL (6 items)**						
Activities unable to perform independently	None	None	Travel independently on public transportation	Travel independently on public transportation	None	None
**Self**-**reported or diagnosed chronic illness (es)**	Cataract	Hypertension	Fractures	Hypertension	None	None
	Gout	Cataract	Osteoporosis	Cataract		
		Thyroid disease				

Note: Katz’s ADL items: (A) bath or shower, (B) dress, (C) toilet, (D) transfer, (E) continence, (F) feed; Lawton & Brody’s IADL items: (A) shop, (B) prepare meals, (C) do the laundry, (D) travel independently on public transportation, (E) operate telephone, (F) handle finances.

The participants were informed about the purpose of the study and consented to participate and be videotaped. All interviews were held in participants’ residences. To protect the anonymity of the respondents, each participant was given a pseudonym in this report.

### Data analysis

Data analysis followed the steps recommended by Braun and Clarke [27]. The interviews were transcribed by a trained research assistant and were then cross-checked with the videos and validated by one of the interviewers. The content of the interview was initially coded independently by two researchers at a general level in order to condense the data into analyzable units. Codes were assigned to segments of transcripts which ranged from a phrase to several sentences based on the pre-selected questions of the semi-structured interview. Initial codes were compared. Then, themes were generated independently and compared. Disagreement in code and theme description and assignment was resolved through constant discussions. A computer program was not used for qualitative analysis due to the small number of participants. To supplement the findings of this study, quantitative findings about the six participants were also checked and used in the analysis.

## Results

### Participants

The age of the six participants (three female, three male) ranged from 98 to108 (Median = 101). All were born in China but had resided in Hong Kong for at least 50 years. Table 1 presents the demographic and health characteristics of the participants.

Four major themes emerged from the six areas of investigation leading to longevity in Hong Kong, namely, (a) *Positive relations with others*, (b) *Possession of memorable positive life events*, (c) *Hope for the future*, and (d) *Positive attitude towards life*.

### Qualitative findings

#### Positive relations with others

In general, all participants, regardless of their living arrangements, reported to have positive relationships with others, especially with younger family members and neighbors. All have maintained frequent contact with their family members. For the three participants who lived alone, they were visited by their children or grandchildren almost every day. The survey data revealed that five of the six participants had “never” felt lonely, and all of them reported to have people to turn to when they wanted to be listened to or helped.

#### Family members

Three participants lived with family members and all reported having very positive relationships with their cohabitants, mostly their grandchildren.

“All of my children and grandchildren respected me.” (Mr. D).

“I have many grand and grand-grandchildren. We two [Mr. D and his wife] gave birth to tens and hundreds of people though we cannot remember all of their names… [Interviewer: So who do you like most and who is socially closer to you?] I chat with ALL of them.” (Mr. D).

“We [Mr. F and his wife] have no argument at all. We do not argue…when things [discussions] seem to escalate, I go back to my room and sleep… We have a very healthy, peaceful, and happy family…” (Mr. F).

#### Neighbors

In Hong Kong, the majority of residents live in apartments/condominiums in high-rise buildings and work for long hours. Hence, it is not surprising that the sense of neighborhood might be inadequate compared with elsewhere. Nonetheless, one of the participants, who lived alone in an old public estate with a considerable population of elderly, underscored the importance of maintaining good relations with neighbors.

“[Interviewer: What is the happiest thing in your life?] Chatting with people. Chatting with people when *Yum Cha* [having *dim sum* dishes and Chinese tea]… Sit on a bench at the ground floor of this building and chat with whoever walks past. That’s the happiest thing in my life.” (Mr. A).

### Possession of memorable positive life events

When they were asked to describe the most memorable positive and negative events in their lives, four participants reported positive events that were work-related and stated that those were the happiest moments for them. One recounted that the happiest event was seeing all of his children being highly educated and working as professors at top universities in the United States. One mentioned that there is no event worth recounting as memorable, but the happiest and most important thing in her life is to be visited by her grandchildren. None of the participants reported any significant negative life events in the interviews. Only one participant described the World War II years as “tough”. All participants recalled only positive life events (most related to their career) and none reported negative life events during the interview. It is noteworthy that, with the supporting evidence from their responses in the quantitative survey, although the majority of participants reported being the happiest when they were working, four of the six participants stated that they were “as happy as when they were young” and all of them “always” felt happy.

“I made and sold tofu until I was 94 years old. I woke up at 4 o’clock every morning. Soaked the soybeans in a gigantic tub. Soaked them for an hour. Use hands to blend them with a stone mill. For six kilograms of soybeans, I could make 200 bowls of tofu. After the soybeans were well blended, I boiled and cooked them in a 4.8 ft-wide wok. [Interviewer: Did anyone help you with this?] I had a partner. We took the tofu to the market by a trolley. I made tofu until I was 94…” (Mr. A).

“The opening of my restaurant. It was a one-floor restaurant [and] around 10,000 square feet [large] with tens of staff. In the whole week of the opening, we invited people to play Chinese lion dance and suona. No one did anything like that in those years. I invented that. The suona was so useful to make the street noisy and attract people’s attention. ” (Mr. D).

“I came to Hong Kong when I was 50+ years old. I had worked as a maid for 33 years since then. I mopped the floor, cleaned toilets, cooked meals, and knitted for my boss. Those were my happy moments. If my boss did not move to China, I did not want to retire.” (Ms. C).

### Hope for the future

Contrary to our prediction, participants mentioned that they had nothing significant to hope for. Instead of asking participants explicitly about their plans for the future, the interviewers used analogs in the probing questions to induce ideas related to the topic. Among participants’ responses, none mentioned their long-term plans. However, some participants mentioned that being able to live to the next day is the only thing they wish for.

“[Interviewer: If God offered you a wish, what would you wish for?] [I hope for] the arrival of tomorrow…or sleeping well.” (Ms. B).

“[Interviewer: Do you plan to do anything that you found you might have missed in your lifetime?] “No. I cannot think too much because what I can do is limited now. The only thing I can do is going to *Yum Cha* day by day as I wake up in the morning.” (Ms. A).

“Nothing…now I have lunch, and then walk to the community center, go back home after that, watch TV, and then go to sleep… I am repeating the same things day by day. What else to think of? If you were living that long life, what can you do? You know, some people are eager to live a great span of life, but they failed… You know this is predetermined.” (Ms. C).

### Positive attitude towards life

We asked participants what their lives were about and what they cared about the most in order to explore their attitudes towards life. Five claimed that they were adventurous, and all of them agreed that life has been beautiful. Their advice to others about longevity was largely related to their attitudes towards life. Here is their advice:

“Don’t think too much…be open-minded to problems…walking and doing exercise after meals are important.” (Mr. A).

“Life is predetermined.” (Ms. B).

“Be kind. Never hurt others.” (Ms. C).

“Hard work. Stay sincere. Honest. Persistence.” (Mr. D).

“God blesses us.” (Ms. E).

“Peace is the most important thing in life. To me, a peaceful family is the most important.” (Mr. F).

## Discussion and conclusions

Studying the survival trajectories of exceptionally long-lived and lived-well individuals enhances our understanding on health and functional capacities of all age groups. This paper reports the first exploratory qualitative study depicting psychological well-being of near-centenarians and centenarians in Hong Kong, a city with one of the world’s highest life expectancies for both genders. Consistent with the literature [16-18,20,28,29], the six oldest-old individuals indicated that having maintained positive relationships with others, possessing a collection of positive life events, and upholding a constellation of salutary attitudes towards life were important to their well-being. It seems that these factors have given a sense of “living life to the fullest with the right people” which has prepared them to be resilient even when challenged in all aspects of life.

Maintaining meaningful social contacts with others and a supportive social network have been found to be vital to sustaining quality of life for old people [30]. Contacts with family, neighbors, friends, and community service providers support longevity by offering valuable social resources which are in line with the conception of “well-adjusted” social life [31]. However, access to these resources may be constrained by living conditions [32]. For example, the Georgia Centenarian Study found that centenarians living in nursing homes reported having significantly fewer social resources compared to those living in either private homes or personal care facilities [33]. Our study only involved individuals who lived in the community. Thus, we could not validate the conclusions drawn by Randall and colleagues concerning elderly persons living in care facilities. Nevertheless, the current findings provide support for the importance of positive relations and supportive social resources for the well-being of near-centenarians and centenarians. Programs that facilitate cognitive functioning and social contacts may be beneficial for enhancing social resources and independence. Good examples include *Senior Odyssey*, which is an adaptation of a program initially developed for grade-school children that focuses on solving novel problems and encourages group collaboration [34], and *the Experience Corps*, which is an intergenerational program in Baltimore, Maryland. The program trains up older adults to help at-risk students in math and reading skills in collaboration with public schools. Through the program, elderly trainers may benefit from spending less time in less engaging activities such as watching television, from cognition-enhancing activities that help preserve their executive functioning and memory, and from establishing social ties with different age groups [35].

Various studies found that positive attitudes towards life and ageing facilitate quality of life among elderly persons. For instance, possessing high self-efficacy or a ‘can-do’ attitude was found to predict good quality of life, along with more commonly anticipated factors such as good physical health and functional ability [36,37]. In the present study, based on participants’ recollection of their life histories, we infer that they tended to have high self-efficacy and a ‘can-do’ attitude in their earlier work life. However, when it came to their current well-being, they tended to emphasize the importance of a ‘let go’ or ‘think less’ attitude and adaptive coping responses towards life as the key to achieving tranquility of the mind [38]. This resonates with previous findings on the importance of adapting to physical limitations for successful ageing [17,31].

In the face of the almost inevitable deterioration in physical functioning and the impending end of life, coping strategies such as acceptance and mental disengagement may be effective in handling fear and insecurities. It is also worth noting that the six participants had shared their perspectives and advice about achieving a long life. Their recommendations covered issues related to spirituality, religiosity, maintaining cognitive astuteness, family, and work ethics. This highlights the need to examine factors of longevity from a multidimensional approach covering biological, psychological, and social aspects, as well as to scrutinize the interactions of different dimensions in the context of elderly people’s daily life and life history. It will be theoretically valuable to collect recommendations on longevity from exceptionally long-lived adults in different societies, as these lay theories could be shaped by factors such as norms about families, cultural ideals, social policies and so forth.

While acknowledging the small sample size and the maximum variation sampling method, work featured very heavily in the recollection of positive life events in all six participants which is in line with one of the qualitative studies suggesting that memories of families and friends, work and work ethic, attitude toward life will enhance longevity directly or indirectly [39]. Also, five of the six participants retired at a relatively advanced age and expressed their enjoyment at work. A late retirement age appears to play an important role in the psychological well-being of aged individuals. In addition to a structured daily routine, the work environment may provide platforms for individuals to excel, to be constructive and productive, and to secure self-worthiness. This finding has significant practical implications for future ageing and public policies. The unprecedented rate of ageing population in many developed countries is expected to result in severe shrinkage of the labor force, and in turn, may threaten economic growth and productivity. If retiring at an older age is prospectively beneficial to elderly persons’ well-being, retaining older individuals in the labor force may appear a desirable and tenable idea. In addition, we share the perspective of Vaupel and Loichinger [40] regarding the benefit of expanding the proportion of labor force among developed countries. In their thesis, they suggested that if a greater share of the population works, multiple beneficial outcomes may follow; for instance (a) reduction in the average number of hours of work per week for all individuals, (b) abatement in youth unemployment rates, (c) increase in quality time spent with children among middle-aged parents as they can “spread out” their time spent on work across a larger proportion of their lifetime, (d) increase in leisure activities for those who are still physically capable.

On the other hand, a sensible alternative is to compensate younger people for working when they are older by allowing them to work fewer hours per week over the whole course of their lives [41]. However, for developing countries, it is acknowledged that relatively more negative outcomes may incur, including reduced income for the majority due to the reduction in working hours. Also, this recommendation may not work for places where the majority of jobs require high physical exertion. The findings of this study may have some contribution to the current discussion about extending the legal age of retirement in Hong Kong and setting up a universal retirement protection scheme.

We acknowledge that the major limitation of this study is its small sample size. However, we would like to stress the difficulties of recruiting extremely healthy near-centenarians and centenarians as the sample of the study. Besides, the collected information seems to skew greatly to the positive side which was not unusual in the centenarian research as some of them were not inclined to talk about the unhappy parts of their life histories [39]. It was noteworthy that our participants did not share much negative information with us during the interviews. This was probably because, first, we specifically told them that we aimed to know how they lived so long and well; second, the participants were reluctant to discuss their concerns with the research team. This is culturally understandable, in that Chinese elders might be relatively conservative and therefore reluctant to discuss openly their weaknesses and worries in front of others out of concern for their own reputation. Chinese culture emphasizes harmony and face-saving (or *mianzi*) [38,42]. *Mianzi* is defined as ‘a social reputation that is highly valued by Chinese’ [42], therefore Chinese may be less likely to disclose their feelings and emotions to others. Previous studies differentiated Chinese communication from North American’s by five communication characteristics which may help explain this limitation: (a) *han xu* or implicit, (b) *ting hua* (listening-centeredness), (c) *ke qi* or politeness, (d) *zi ji ren* (insider-oriented) and (e) *mianzi* or face-directed [43,44]. During the interviews, *implicit* might play so as to limit responses to an emotionally controllable level. *Listening*-*centeredness* and *Polite* might encourage the elderly to answer more positively to entertain the interviewers. *Insider*-*oriented* may help explain why they refrain from full and free expression, because the interviewers might not be treated as an “Insider”. Third, in order to protect face, they might hesitate to share life events that might diminish their reputation with the interviewers.

Regardless of these limitations, studying the successfully aged not only allows us to better understand the process of ‘survivorship’ in positive and successful ageing, but also reminds us of what needs to be planned and implemented in our rapidly ageing society, a phenomenon so far unseen in the history of mankind.

### Endnote

^a^The U3A project is sponsored by the Hong Kong Electric Centenary Trust and coordinated by the Hong Kong Council of Social Service (HKCSS) to promote active ageing and life-long learning among the city’s retired population. Its network covers 16 elderly service agencies in Hong Kong. The agencies receive funding to set up self-learning network/centers that provide courses conducted by older persons for the older persons. The webpage is http://www.u3a.org.hk/.

### Appendix A

*Semi*-*structured open*-*ended questions*

Social network contact and family support

• How frequently do you have contact with your family members (ask specifically for each individuals)?

• Please evaluate the assistance received from family members in the form of a) household items; b) money; c) emotional support; and d) informational support.

• Please list up to FIVE individuals who could be considered “significant others” (can include friends, relatives, neighbors, helping professionals, or anyone else), regardless of how often they have contact with you, in your life?

• After the list is generated, ASK: a) what was the nature of the relationship; b) how often do you meet, and by what means; and c) how does he/she affect your life now?

Life events

• Life events (positive and negative) happened a) in the past year; and b) in your lifetime, that are most memorable to you? How do they affect you?

Life attitude

• Many people have a lot of attitudes about many things, e.g. work, love, etc. What is your attitude about life? Probe if not understood (e.g. What is LIFE to you? What do care most about in life etc? Do you spend a lot of time doing things that are productive? Do you feel good because you do activities are meaningful and purposeful).

Hope

• What do you hope for now?

Happiness

• Are you as happy as when you were younger?

• Do you think your life is the happiest now?

• Do you think now are the best years of your life?

• Can you tell me why?

Congruence

• As you look back on your life, are you satisfied?

• If you could change your life, what aspects would you change?

Personality

• How would you describe your personality?

*Motto and suggestions to others regarding living a long life*.

## Competing interests

The authors declare that they have no competing interests.

## Authors’ contributions

SLKC is the Principal Investigator of the project and PWCW, YMAL, MYGC, and WMC are the Co-investigators. SLKC, PWCW, and BHPL conducted the interviews. PWCW and NCFK conducted data analysis. PWCW, BHPL, NCFK, and SLKC wrote up the manuscript. All authors commented on and approved the manuscript.

## Pre-publication history

The pre-publication history for this paper can be accessed here:

http://www.biomedcentral.com/1471-2318/14/63/prepub
